# Activation of Apoptosis in a βB1-CTGF Transgenic Mouse Model

**DOI:** 10.3390/ijms22041997

**Published:** 2021-02-17

**Authors:** Maximilian Weiss, Sabrina Reinehr, Ana M. Mueller-Buehl, Johanna D. Doerner, Rudolf Fuchshofer, Gesa Stute, H. Burkhard Dick, Stephanie C. Joachim

**Affiliations:** 1 Experimental Eye Research Institute, University Eye Hospital, Ruhr-University Bochum, In der Schornau 23-25, 44892 Bochum, Germany; Maximilian.Weiss@rub.de (M.W.); sabrina.reinehr@rub.de (S.R.); Ana.Mueller-Buehl@rub.de (A.M.M.-B.); jd@laser-24.de (J.D.D.); gesa.stute@rub.de (G.S.); Burkhard.Dick@kk-bochum.de (H.B.D.); 2 Institute of Human Anatomy and Embryology, University Regensburg, Universitätsstraße 31, 93053 Regensburg, Germany; rudolf.fuchshofer@vkl.uni-regensburg.de

**Keywords:** βB1-CTGF, primary open-angle glaucoma, apoptosis, caspase 3, neurofilament H, Bax/Bcl2, synapse

## Abstract

To reveal the pathomechanisms of glaucoma, a common cause of blindness, suitable animal models are needed. As previously shown, retinal ganglion cell and optic nerve degeneration occur in βB1-CTGF mice. Here, we aimed to determine possible apoptotic mechanisms and degeneration of different retinal cells. Hence, retinae were processed for immunohistology (*n* = 5–9/group) and quantitative real-time PCR analysis (*n* = 5–7/group) in 5- and 10-week-old βB1-CTGF and wildtype controls. We noted significantly more cleaved caspase 3^+^ cells in βB1-CTGF retinae at 5 (*p* = 0.005) and 10 weeks (*p* = 0.02), and a significant upregulation of *Casp3* and *Bax/Bcl2* mRNA levels (*p* < 0.05). Furthermore, more terminal deoxynucleotidyl transferase-mediated dUTP nick end labeling (TUNEL^+^) cells were detected in transgenic mice at 5 (*p* = 0.03) and 10 weeks (*p* = 0.02). Neurofilament H staining (*p* = 0.01) as well as *Nefh* (*p* = 0.02) and *Tubb3* (*p* = 0.009) mRNA levels were significantly decreased at 10 weeks. GABAergic synapse intensity was lower at 5 weeks, while no alterations were noted at 10 weeks. The glutamatergic synapse intensity was decreased at 5 (*p* = 0.007) and 10 weeks (*p* = 0.01). No changes were observed for bipolar cells, photoreceptors, and macroglia. We conclude that apoptotic processes and synapse loss precede neuronal death in this model. This slow progression rate makes the βB1-CTGF mice a suitable model to study primary open-angle glaucoma.

## 1. Introduction

Glaucoma is a leading cause of blindness worldwide [1]. It is a chronic, progressive neuropathy in which apoptosis of retinal ganglion cells (RGCs) and loss of optic nerve axons result in structural and functional deficits, such as visual field loss [2,3]. The main and currently only modifiable risk factor is the high intraocular pressure (IOP). At present, the only treatment with evidence in preserving visual function is the reduction in IOP [3,4,5]. This is associated with reduced disease progression but is not able to revert damage [6]. This poses a problem, since glaucoma is often diagnosed at advanced stages, when about 30% of RGCs are already lost [4]. Therefore, allowing for diagnoses at earlier stages could have a substantial impact on the glaucoma therapy. To develop new diagnosis tools as well as novel therapy options, a reliable animal model for primary open-angle glaucoma (POAG) is necessary. In POAG, the increased IOP is caused by a high resistance to aqueous humor outflow in the trabecular meshwork [7,8]. The transgenic glaucoma model of the connective tissue growth factor (βB1-CTGF) mouse simulates these pathomechanisms without any surgical interventions. In this model, the lens-specific overexpression of CTGF in mice is responsible for changes in the cytoskeleton and the extracellular matrix in the trabecular meshwork. In 2012, Junglas et al. demonstrated that 4-week-old βB1-CTGF mice with a FVB/NxCD1 background develop an elevated IOP accompanied by a loss of optic nerve axons [7]. To investigate whether glaucoma-like damage also occurs in the retinae of these animals, we performed *in vivo* and *in vitro* analyses of βB1-CTGF mice. The animals in our study were backcrossed and had a CD1 background, since it is known that this strain is more susceptible to IOP changes [9,10]. The transgenic mice developed a significantly increased IOP at 15 weeks of age, while at 5 and 10 weeks, the IOP remained unaltered. In addition, we could note a loss of retinal function, a decline in RGC and cone bipolar cell numbers, as well as reactive macroglia at the age of 15 weeks. Different time courses between both studies could be explained by using different background strains [11]. Nonetheless, little is known about the early retinal changes in βB1-CTGF mice. Therefore, further characterization of this model is needed.

The aim of this study is to investigate possible apoptotic processes and changes in retinal cells and synapses. We show early apoptosis starting in 5-week-old transgenic mice, leading to a loss of neurofilament H at 10 weeks of age. This was accompanied by a loss of glutamatergic synapses in transgenic animals.

## 2. Results

### 2.1. Loss of Neurofilament H at 10 Weeks

**Figure 1:**
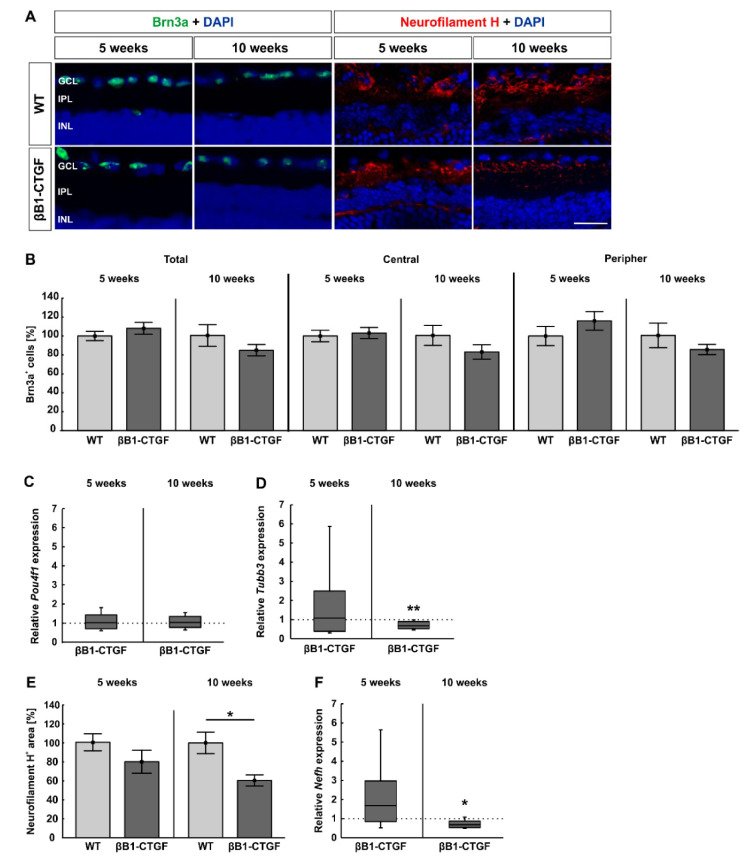
Loss of neurofilament H. (**A**) Retinae were stained with antibodies against Brn-3a (green) and neurofilament H (red) at 5 and 10 weeks (n = 5–8/group). Cell nuclei were visualized with 4′,6 diamidino-2-phenylindole (DAPI) (blue). (**B**) The number of Brn3a^+^ cells remained unaltered in βB1-CTGF retinae in comparison to wildtype (WT) at 5 and 10 weeks of age in the total as well as in the central and peripheral retina. (**C**) RT-qPCR analyses were performed regarding the mRNA expression levels of the retinal ganglion cell (RGC) marker *Pou4f1* at both ages (n = 5–7/group). In accordance with the immunohistological results, no alterations in *Pou4f1* expression levels were noted in βB1-CTGF animals at the age of 5 and 10 weeks compared to in WT. (**D**) RT-qPCR analyses of *Tubb3* were performed in 5- and 10-week-old βB1-CTGF and WT mice (n = 5/group). At 5 weeks, no changes were seen in *Tubb3* mRNA expression levels. In contrast, a significant downregulation of *Tubb3* mRNA was revealed in transgenic mice at 10 weeks of age (*p* = 0.09). (**E**) At 5 weeks, the staining area of neurofilament H remained unchanged in transgenic and WT animals. In contrast, a significant decrease in the neurofilament H^+^ area was detected in βB1-CTGF mice (*p* = 0.01). (**F**) The mRNA levels of *Nefh* (n = 5/group) were not altered in 5-week-old transgenic mice. However, a significant downregulation of *Nefh* mRNA levels was observed in βB1-CTGF mice at 10 weeks (*p* = 0.02). Abbreviations: GCL = ganglion cell layer, IPL = inner plexiform layer, INL = inner nuclear layer. Values are mean ± SEM for immunohistology and median ± quartile + maximum/minimum for RT-qPCR. The dotted lines in C, D, and F represent the relative expression levels of the WT group. Scale bar: 20 μm. * *p* < 0.05, ** *p* < 0.01.

In order to analyze the number of RGCs in 5- and 10-week-old βB1-CTGF mice, immunohistological staining with antibodies against Brn3a and neurofilament H was performed (Figure 1A). We could see a comparable total number of Brn-3a^+^ cells in 5-week-old βB1-CTGF (108.18 ± 6.24%) and wildtype (WT) mice (100.00 ± 4.81%; *p* = 0.32). At 10 weeks of age, the βB1-CTGF (84.51 ± 6.00%) and the WT groups (100.00 ± 11.42%; *p* = 0.25) revealed similar RGC counts (Figure 1B). The RGC number in the central part of the retina was comparable in 5-week-old βB1-CTGF (103.27 ± 5.93%) and WT animals (100.00 ± 6.03%; *p* = 0.28). This could also be noted at 10 weeks (WT: 100.00 ± 10.58%; βB1-CTGF: 82.64 ± 7.63%; *p* = 0.20; Figure 1B). In the peripheral parts, the number of Brn3a^+^ RGCs did not differ between transgenic (116.08 ± 9.85%) and WT mice (100.00 ± 10.15%; *p* = 0.71) at 5 weeks. In addition, at 10 weeks, similar RGC counts were detected in βB1-CTGF (85.24 ± 5.33%) and WT animals (100.00 ± 12.91%; *p* = 0.31; Figure 1B).

Furthermore, the RT-qPCR analyses showed similar mRNA expression levels of the RGC marker *Pou4f1* in βB1-CTGF mice compared to in WT mice at 5 (1.02-fold expression; *p* = 0.09) and 10 weeks (1.04-fold expression; *p* = 0.75; Figure 1C).

Regarding *Tubb3* mRNA levels, a neuronal marker, no alterations were noted between βB1-CTGF and WT mice (1.08-fold expression; *p* = 0.86) at 5 weeks. 10-week-old transgenic animals already had significantly downregulated *Tubb3* mRNA levels (0.67-fold expression; *p* = 0.009; Figure 1D).

Regarding neurofilament H, at 5 weeks, the stained area was comparable in βB1-CTGF (79.64 ± 12.08%) and WT animals (100.00 ± 8.92%; *p* = 0.2; Figure 1E). Then, a significant decrease in the neurofilament H^+^ area was revealed in transgenic mice (60.42 ± 5.92%) compared to in WT ones (100.00 ± 11.25%; *p* = 0.01) at 10 weeks of age (Figure 1E).

No alterations in the expression levels of *Nefh* could be noted between βB1-CTGF and WT mice at 5 weeks (1.68-fold expression; *p* = 0.15). However, in accordance with the immunohistological findings, a significant downregulation of *Nefh* mRNA was observed in 10-week-old transgenic animals (0.69-fold expression; *p* = 0.02; Figure 1F).

These results reveal a loss of neurofilament H in βB1-CTGF mice at the age of 10 weeks.

### 2.2. Increased Number of Apoptotic Retinal Ganglion Cells

**Figure 2:**
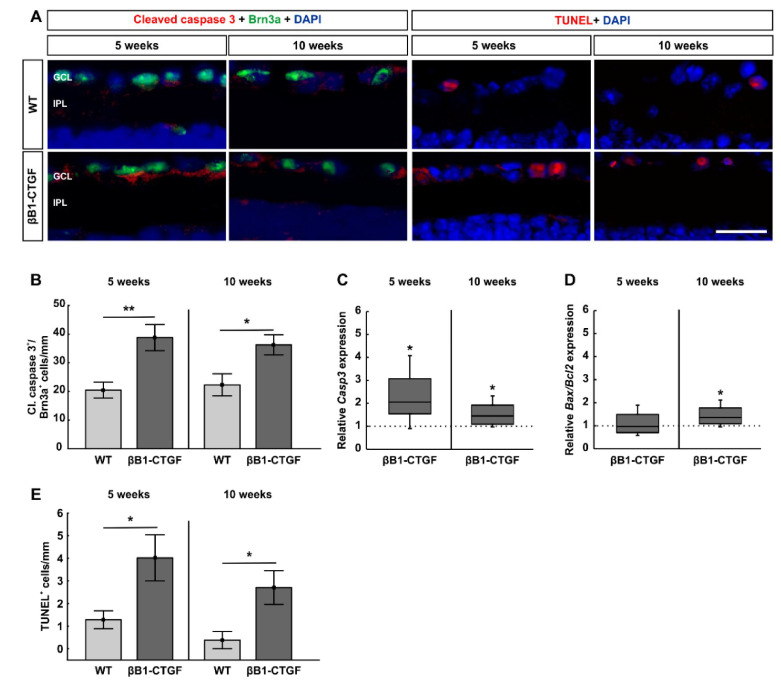
Increased apoptosis rate in retinal ganglion cells. (**A**) Retinae were stained with the apoptosis marker cleaved caspase 3 (red) in combination with Brn3a (green) at 5 and 10 weeks (n = 7–8/group). Furthermore, apoptotic cells were visualized using terminal deoxynucleotidyl transferase-mediated dUTP nick end labeling (TUNEL) staining (n = 5/group). Cell nuclei were visualized with DAPI (blue). (**B**) Significantly more cleaved caspase 3^+^ RGCs were revealed in βB1-CTGF mice compared to in WT animals at 5 (*p* = 0.005) and 10 weeks (*p* = 0.02). (**C**) The mRNA expression levels of *Casp3* were upregulated at 5 (*p* = 0.02) and 10 weeks (*p* = 0.03). (**D**) At 5 weeks, the mRNA expression levels of *Bax/Bcl2* were not altered in βB1-CTGF mice. In contrast, a significant upregulation of *Bax/Bcl2* levels was noted in 10-week-old transgenic animals (*p* = 0.04). (**E**) A higher number of TUNEL^+^ apoptotic cells in the ganglion cell layer was reveled in 5- (*p* = 0.03) and 10-week-old βB1-CTGF mice compared to in WT (*p* = 0.02). Abbreviations: GCL = ganglion cell layer, IPL = inner plexiform layer. Values are mean ± SEM for immunohistology and median ± quartile + maximum/minimum for RT-qPCR. The dotted lines in C and D represent the relative expression levels of the WT group. Scale bar: 20 μm. * *p* < 0.05, ** *p* < 0.01.

To detect possible apoptosis of RGCs, retinal cross sections were co-stained with an anti-cleaved caspase 3 antibody and anti-Brn3a (Figure 2A). At 5 weeks of age, we already noted significantly more cleaved caspase 3^+^ RGCs in βB1-CTGF mice (38.81 ± 4.58 cells/mm) compared to in WT mice (20.47 ± 2.76 cell/mm; *p* = 0.005). We still observed significantly more cleaved caspase 3^+^ RGCs in βB1-CTGF retinae (36.26 ± 3.53 cells/mm) compared to in WT retinae (22.29 ± 3.85 cells/mm; *p* = 0.02) at 10 weeks (Figure 2B).

Additionally, the mRNA expression levels of *Casp3* were significantly increased in transgenic mice at 5 (2.06-fold expression; *p* = 0.02) and 10 weeks of age (1.45-fold expression; *p* = 0.03; Figure 2C). This is in agreement with the immunohistological data.

Furthermore, we analyzed the expression levels of *Bax/Bcl2* mRNA. At 5 weeks, the mRNA levels of *Bax/Bcl2* were comparable between βB1-CTGF and WT mice (0.97-fold expression; *p* = 0.9). Then, a significant upregulation of *Bax/Bcl2* mRNA was noted in 10-week-old transgenic mice (1.36-fold expression; *p* = 0.04, Figure 2D).

In addition, terminal deoxynucleotidyl transferase-mediated dUTP nick end labeling (TUNEL) staining, an established method to detect DNA fragmentation, such as in apoptosis, was performed on retinal cross sections of βB1-CTGF and WT animals to further investigate cell death in this model (Figure 2A). A significantly higher number of TUNEL^+^ cells in the ganglion cell layer of βB1-CTGF mice was detected (4.02 ± 1.02 cells/mm) compared to that in WT ones (1.28 ± 0.40 cells/mm; *p* = 0.03) at 5 weeks. Also, at 10 weeks, an even higher number of TUNEL^+^ cells could be monitored in transgenic animals (2.70 ± 1.49 cells/mm) in comparison to WT mice (0.38 ± 0.38 cells/mm; *p* = 0.02; Figure 2E)

To summarize, apoptotic mechanisms are notable in 5-week-old βB1-CTGF mice and seem to play a major role in subsequent cell death.

### 2.3. Loss of Synapses

**Figure 3:**
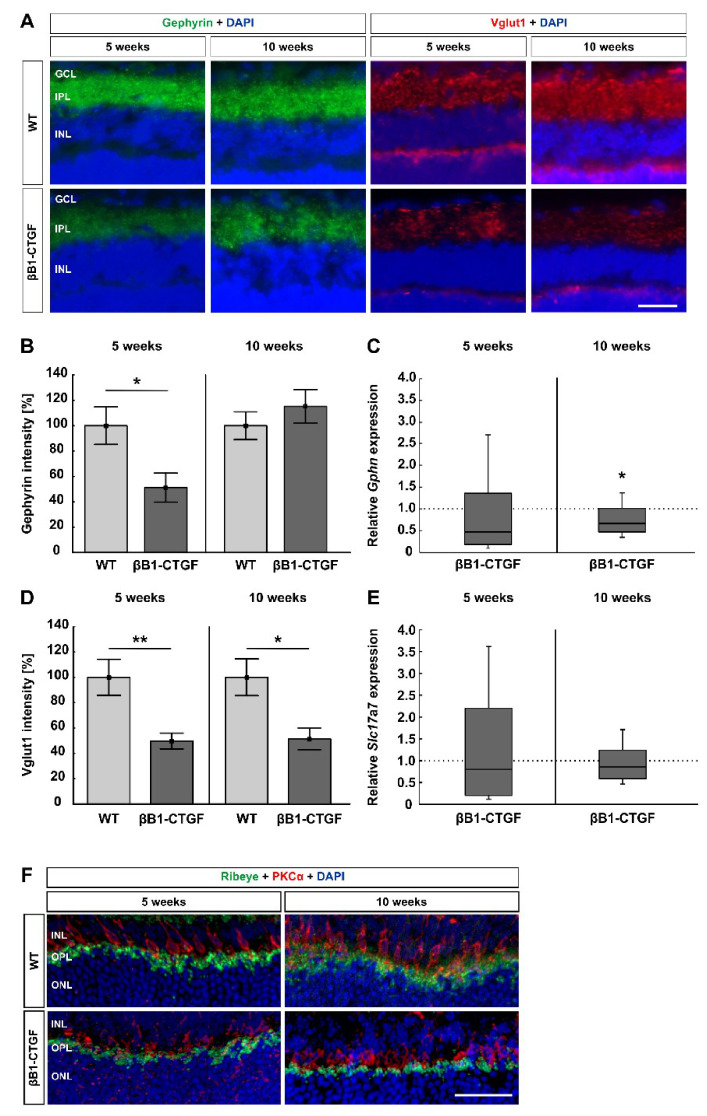
Loss of synapses. (**A**) GABAergic synapses were labeled using gephyrin (green) and glutamatergic synapses were stained with Vglut1 (red), while cell nuclei were counterstained with DAPI (blue) at 5 and 10 weeks of age (n = 7–8/group). (**B**) At 5 weeks of age, a significantly decreased gephyrin^+^ intensity was revealed in βB1-CTGF mice (*p* = 0.02). At 10 weeks, the staining intensity reverted to the WT level. (**C**) The mRNA expression levels of *Gphn* (n = 5–7/group) showed no changes at 5 weeks, but a significant downregulation was shown in 10-week-old βB1-CTGF mice (*p* = 0.02). (**D**) Regarding Vglut1 staining, significantly decreased immunoreactivity was noted in transgenic mice at 5 (*p* = 0.007) and 10 weeks of age (*p* = 0.01). (**E**) RT-qPCR analyses detected no alterations in *Slc17a7* (Vglut1) mRNA expression levels at both points in time (n = 5–7/group). (**F**) Exemplary, retinal cross sections were labeled with antibodies against ribeye (ribbon synapses; green) and PKCα (rod bipolar cells; red). DAPI counterstained cell nuclei (blue). At 5 and 10 weeks, βB1-CTGF and WT mice displayed distinct co-staining of both markers in the inner plexiform and outer plexiform layer. In the WT animals at both ages, the co-staining was more intense compared to transgenic mice. Abbreviations: GCL = ganglion cell layer, IPL = inner plexiform layer, INL = inner nuclear layer, OPL = outer plexiform layer, ONL = outer nuclear layer. Values are mean ± SEM for immunohistology and median ± quartile + maximum/minimum for RT-qPCR. The dotted lines in C and E represent the relative expression levels of the WT group. Scale bars: 20 μm. * *p* < 0.05, ** *p* < 0.01.

Alterations in synapses were detected by staining retinae with anti-gephyrin (GABAergic synapses) and anti-Vglut1 (glutamatergic synapses; Figure 3A). The gephyrin^+^ area was significantly decreased in βB1-CTGF animals (51.21 ± 11.42%) compared to in WT retinae (100.00 ± 14.78%; *p* = 0.02) at 5 weeks. At 10 weeks, no changes in the gephyrin^+^ area were noted in transgenic mice (115.22 ± 13.10%) when compared to in WT ones (100.00 ± 10.91%; *p* = 0.4; Figure 3B).

The *Gphn* mRNA analyses revealed no changes at 5 weeks (0.47-fold expression; *p* = 0.2). At 10 weeks, a significant downregulation of *Gphn* mRNA levels was noted in βB1-CTGF retinae (0.67-fold expression; *p* = 0.02; Figure 3C).

At 5 weeks, a significantly lower Vglut1 intensity was detectable in βB1-CTGF retinae (49.72 ± 6.22%) compared to in WT animals (100.00 ± 14.20%; *p* = 0.007). Also, at 10 weeks, a significant decrease in the Vglut1 intensity was seen in the βB1-CTGF mice (51.34 ± 8.71%) compared to in the WT ones (100.00 ± 14.50%; *p* = 0.01; Figure 3D).

The mRNA expression of *Slc17a7* (encodes Vglut1 in mice) did not differ in βB1-CTGF retinae at 5 (0.8-fold expression; *p* = 0.7) and 10 weeks (0.9-fold expression; *p* = 0.3; Figure 3E).

Furthermore, ribbon synapses were visualized with an anti-ribeye antibody and co-stained with an antibody against PKCα (rod bipolar cells; Figure 3F). We could show co-staining of ribeye^+^ ribbon synapses and PKCα^+^ rod bipolar cells in both groups in the inner nuclear and outer plexiform layers. In WT animals at 5 and 10 weeks of age, co-staining of ribeye and PKCα was more intense compared to the corresponding βB1-CTGF mice. We assumed alterations in ribbon synapses at early stages in this transgenic mouse model.

These results suggest an early loss of synapses, before a significant cell loss can be observed in βB1-CTGF mice.

### 2.4. Similar Bipolar Cell Numbers

**Figure 4:**
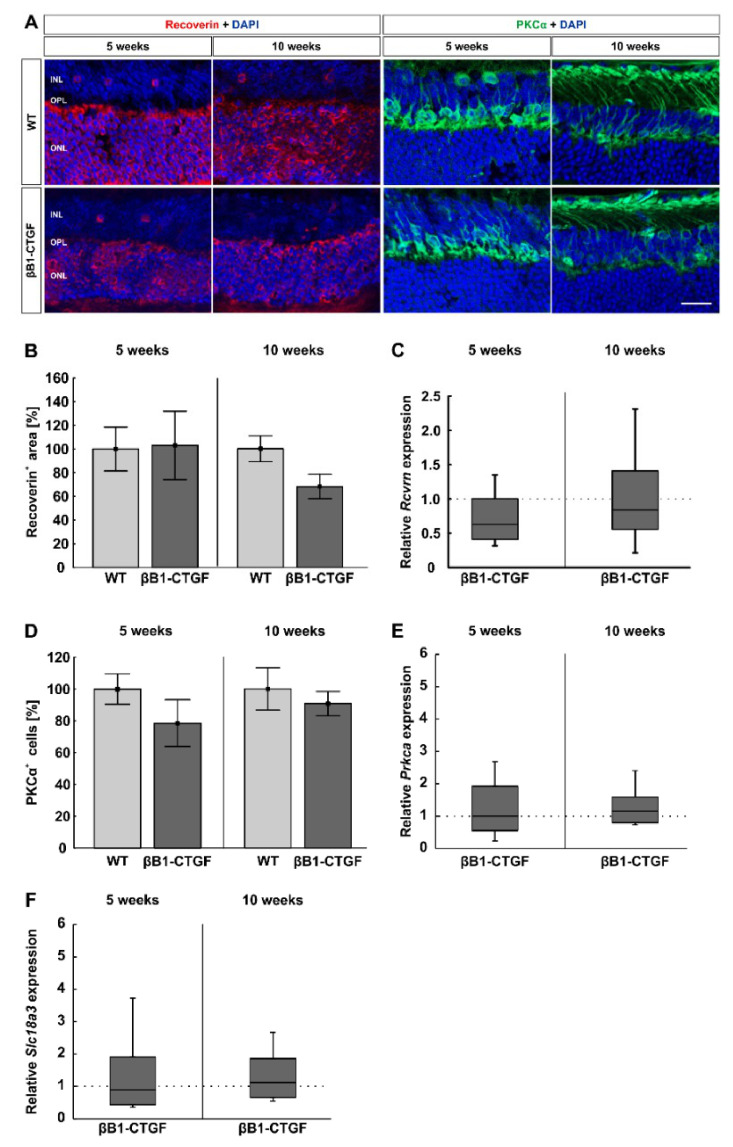
No alterations in the number of bipolar cells. (**A**) Cone bipolar cells were evaluated by using recoverin (red) and rod bipolar cells using PKCα staining (green; n = 7–9/group). Cell nuclei were labeled with DAPI (blue). (**B**) The analysis of the recoverin^+^ area revealed no significant difference in staining area in βB1-CTGF animals at 5 weeks. At the age of 10 weeks, there was a trend towards smaller cone, bipolar cell areas in βB1-CTGF mice compared to in WT mice (*p* = 0.052). (**C**) The *Rcvrn* mRNA expression levels were comparable between βB1-CTGF and WT animals at both ages (n = 5–7/group). (**D**) PKCα^+^ cell numbers were not altered in βB1-CTGF retinae compared to in WT at 5 and 10 weeks. (**E**) RT-qPCR analyses of *Prkca* mRNA expression levels showed no alterations at 5 and 10 weeks of age. (**F**) Additionally, the mRNA levels of *Slc18a3* (encoding for the vesicular acetylcholine transporter) were not altered in 5- and 10-week-old βB1-CTGF animals. Abbreviations: INL = inner nuclear layer, OPL = outer plexiform layer, ONL = outer nuclear layer. Values are mean ± SEM for immunohistology and median ± quartile + maximum/minimum for RT-qPCR. The dotted lines in C, E, and F represent the relative expression levels of the WT group. Scale bar: 20 µm.

To evaluate the number of cone and rod bipolar cells, retinal cross sections were labeled with anti-PKCα (rod bipolar cells) and anti-recoverin (cone bipolar cells; Figure 4A). At 5 weeks, the cone bipolar cell number was similar in βB1-CTGF mice (103.09 ± 28.88%) and WT mice (100.00 ± 18.58%; *p* = 0.93). At 10 weeks, there were comparable recoverin^+^ cone bipolar cells in βB1-CTGF mice (67.39 ± 10.49%) compared to in WT mice (100.00 ± 11.15%; *p* = 0.052; Figure 4B).

The RT-qPCR analyses displayed similar *Rcvrn* mRNA levels in βB1-CTGF and WT animals at 5 (0.68-fold expression; *p* = 0.61) and 10 weeks (0.84-fold expression; *p* = 0.48; Figure 4C).

The number of rod bipolar cells was comparable between βB1-CTGF mice (78.56 ± 14.69%) and WT animals (100.00 ± 9.60%; *p* = 0.23) at 5 weeks. Additionally, at 10 weeks of age, no changes in the number of PKCα^+^ cells were detected in βB1-CTGF retinae (90.80 ± 7.66%) compared to in WT ones (100.00 ± 13.27%; *p* = 0.57; Figure 4D).

RT-qPCR analyses of *Prkca* mRNA showed no alterations in transgenic mice compared to in WT retinae at 5 (1.00-fold expression; *p* = 0.99) and 10 weeks of age (1.16-fold expression; *p* = 0.45; Figure 4E).

Additionally, the mRNA levels of *Slc18a3* (encoding for the vesicular acetylcholine transporter) were not altered in 5- (0.89-fold expression; *p* = 0.79) and 10-week-old βB1-CTGF animals (1.13-fold expression; *p* = 0.59; Figure 4F).

In summary, cone and rod bipolar cells were not affected in 5- and 10-weeks-old transgenic mice compared to in WT ones.

### 2.5. No Effects on Photoreceptors

**Figure 5:**
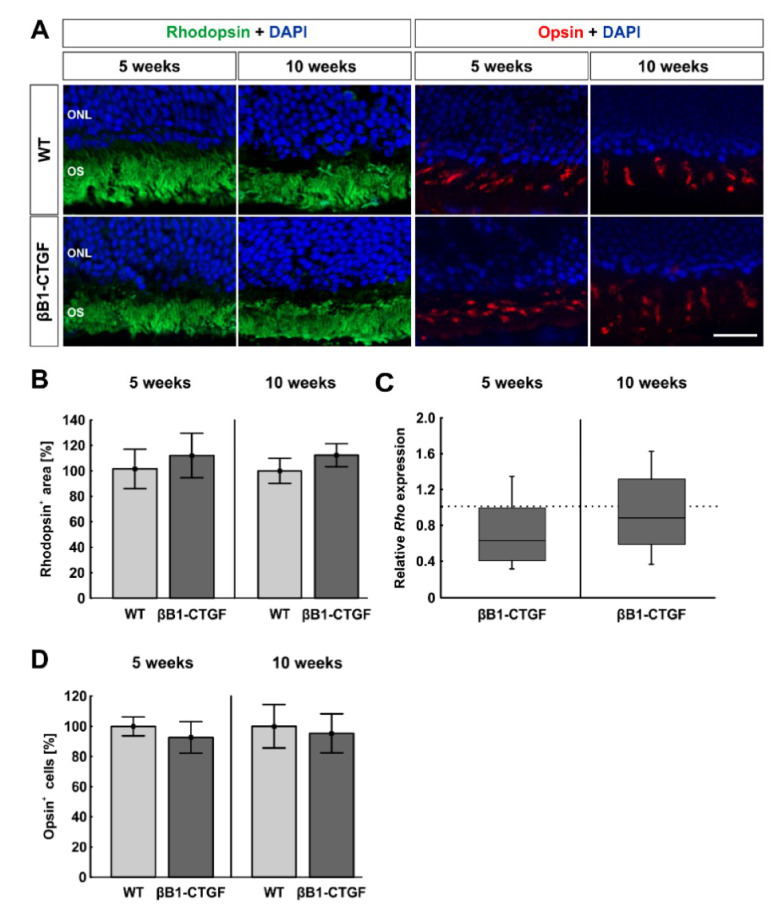
Photoreceptors remain unaffected. (**A**) Rhodopsin (rods, green) and opsin (L-cones, red) staining of retinal sections were performed to evaluate photoreceptors (n = 7–9/group). DAPI was added to visualize cell nuclei (blue). (**B**) Comparable rhodopsin^+^ areas were detected in both groups at 5 and 10 weeks of age. (**C**) The RT-qPCR results showed no alteration in *Rho* mRNA expression levels in βB1-CTGF mice at the age of 5 and 10 weeks (n = 5–7/group). (**D**) The number of opsin^+^ cells remained unaltered in βB1-CTGF animals compared to in WT at both ages. Abbreviations: ONL = outer nuclear layer, OS = outer segment. Values are mean ± SEM for immunohistology and median ± quartile + maximum/minimum for RT-qPCR. The dotted line in C represents the relative expression level of the WT group. Scale bar: 20 µm.

To analyze possible alterations in photoreceptors, the rods were labeled with anti-rhodopsin and L-cones with anti-opsin (Figure 5A). No significant difference in rods could be noted at 5 weeks of age (βB1-CTGF: 110.31 ± 17.21%; WT: 100.00 ± 15.26%; *p* = 0.66). At 10 weeks, the rhodopsin^+^ area remained unchanged in βB1-CTGF retinae (112.33 ± 9.09%) compared to in WT animals (100.00 ± 9.90%; *p* = 0.38; Figure 5).

This was confirmed by RT-qPCR analysis, where no significant downregulation of *Rho* mRNA levels was noted in βB1-CTGF animals at both points in time (5 weeks: 0.63-fold expression; *p* = 0.06; 10 weeks: 0.89-fold expression; *p* = 0.46; Figure 5C).

The number of L-cones was comparable in βB1-CTGF retinae (5 weeks: 92.68 ± 10.45%; 10 weeks: 95.30 ± 12.91%) and WT retinae (5 weeks: 100.00 ± 6.30%; *p* = 0.56; 10 weeks: 100.00 ± 14.36%; *p* = 0.81) at 5 and 10 weeks (Figure 5D).

These results indicate that photoreceptors are not affected in βB1-CTGF mice at 5 and 10 weeks of age.

### 2.6. No Alteration in Macroglia

**Figure 6:**
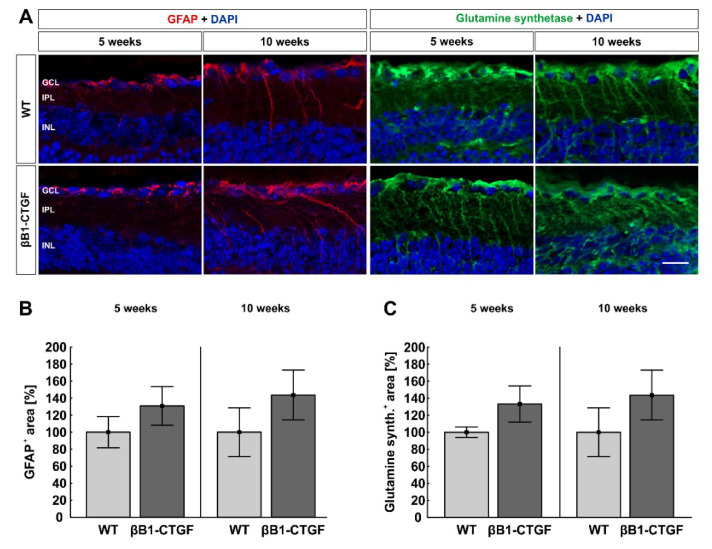
No macroglia reaction. (**A**) Macroglia were analyzed using GFAP (red) and glutamine synthetase (green) at 5 and 10 weeks (n = 7–9/group). DAPI (blue) visualized cell nuclei. (**B**) The GFAP area analysis showed a similar GFAP signal area in βB1-CTGF retinae and WT retinae at 5 and 10 weeks. (**C**) Regarding glutamine synthetase, we observed no difference in the staining area in βB1-CTGF mice compared to in WT at both ages. Abbreviations: GCL = ganglion cell layer, IPL = inner plexiform layer, INL = inner nuclear layer. Values are mean ± SEM. Scale bar: 20 µm.

To examine a possible macrogliosis, retinal cross sections were labeled with anti-GFAP and anti-glutamine synthetase (Figure 6A). Analysis of the GFAP^+^ area indicated a similar GFAP signal area in βB1-CTGF (130.88 ± 22.60%) and WT retinae (100.00 ± 18.35%; *p* = 0.31) at 5 weeks. Additionally, at 10 weeks, no alterations were noted in βB1-CTGF mice (142.47 ± 27.66%) compared to in WT (100.00 ± 27.99%; *p* = 0.30; Figure 6).

No significant difference between βB1-CTGF mice (5 weeks: 133.23 ± 21.25%; 10 weeks: 143.64 ± 29.27%) and WT mice (5 weeks: 100.00 ± 6.26%; *p* = 0.16; 10 weeks: 100.00 ± 28.66%; *p* = 0.30) could be detected with regard to glutamine synthetase^+^ signal area at both ages (Figure 6C).

In summary, at 5 and 10 weeks of age, no macroglia alterations could be observed in βB1-CTGF animals. Later, at 15 weeks, an increased macroglia activation was noted in transgenic mice [11].

### 2.7. No Functional or Morphological alterations

Electroretinogram (ERG) analyses were performed in 5- and 10-week-old animals. The recordings at a light intensity of 10 cd.s/m^2^ are pictured. The electrical output of the photoreceptors is represented by the a-wave amplitude. At 5 weeks, no difference between βB1-CTGF and WT mice could be noted at all light intensities (*p* > 0.05). At 10 weeks, the a-wave amplitude was also comparable between βB1-CTGF and WT mice at all light intensities (*p* > 0.05; Supplementary Figure S1A). The b-wave amplitude represents the electrical output of the inner nuclear layers. At all light intensities, we noted no significant change in the b-wave amplitude in βB1-CTGF mice compared to WT mice at 5 weeks and 10 weeks (*p* > 0.05; Supplementary Figure S1B).

To analyze the thickness of the retina, spectral domain ocular coherence tomographies (SD-OCTs) were performed in βB1-CTGF and WT animals at 5 and 10 weeks of age (Supplementary Figure S1C). The total retinal thickness was comparable between βB1-CTGF (92.42±8.34 %) and WT mice (100.00±2.02 %; *p* = 0.42) at 5 weeks. At 10 weeks, there was also no significant difference between βB1-CTGF (99.06±2.51 %) and WT retinae (100.00±2.13 %; *p* = 0.78) in the total thickness. No differences were noted regarding the central retinal thickness in 5-week-old animals (WT: 100.00±5.29 %; βB1-CTGF: 95.76±7.80 %; *p* = 0.67). Also, at 10 weeks, the central retinal thickness did not differ between transgenic (108.06±3.48 %) and WT mice (100.00±7.58 %; *p* = 0.40). Furthermore, measurements of the peripheral part of the retina showed no alterations at 5 (WT: 100.00±0.98 %; βB1-CTGF: 91.03±9.03%; *p* = 0.37) and 10 weeks (WT: 100.00±2.01 %; βB1-CTGF: 96.95±2.95%; *p* = 0.39; Supplementary Figure S1D).

Additionally, the ganglion cell complex, including nerve fiber layer, ganglion cell layer, and inner plexiform layer, was analyzed. The total retinal thickness of the ganglion cell complex did not differ between transgenic mice (95.18±4.42 %) and WT animals (100.00±1.72 %; *p* = 0.40) at 5 weeks of age. Also, at 10 weeks, no changes were detectable in βB1-CTGF animals (99.39±2.18 %) compared to WT (100.00±0.98 %; *p* = 0.81). The analysis of the central ganglion cell complex showed no differences in βB1-CTGF (98.34±4.60 %) and WT animals (100.00±2.69 %; *p* = 0.77) at 5 weeks. Also, at 10 weeks, the central thickness remained unchanged between transgenic (103.87±2.45 %) and WT mice (100.00±1.80 %; *p* = 0.22). Moreover, the peripheral ganglion cell complex thickness was comparable in 5-week-old βB1-CTGF animals (86.47±6.94 %) and WT mice (100.00±2.19 %; *p* = 0.10). At 10 weeks, the peripheral thickness remained unaltered between transgenic (95.81±2.75 %) and WT animals (100.00±1.26 %; *p* = 0.16; Supplementary Figure S1E).

These results show that neither the retinal function or the thickness was affected in 5- and 10-week-old βB1.CTGF mice.

## 3. Discussion

Glaucoma affects approximately 60 million people worldwide, making it the second most common cause of irreversible blindness [12]. To understand the underlying pathomechanisms, appropriate models are required. Currently, the most widely used glaucoma model is the DBA/2J mouse. However, DBA/2J mice develop a secondary angle-closure glaucoma initiated by iris atrophy, which occurs only in 1–1.5% of all glaucoma patients [4,13,14]. In most animal models of POAG, the IOP is increased through surgical interventions [15,16,17,18]. Often, IOP is highly elevated and the manipulations lead to local inflammations and are therefore detrimental for, e.g., analyses of the immune response. Hence, it would be beneficial to have a mouse model that reflects the pathogenesis of POAG as closely as possible. It is known that an important difference between glaucomatous and healthy eyes is the higher amount of active transforming growth factor in the extracellular matrix [12,19,20]. Due to this fact, Junglas et al. generated a transgenic mouse model that simulates POAG by overexpressing CTGF, leading to optic nerve degeneration [7]. These mice had a mixed FVB/NxCD1 background and were subsequently backcrossed into a pure CD1 background. This was done since the optic nerves of CD1 mice are more susceptible for IOP alterations [9,10]. In these mice, we observed in a previous study that the βB1-CTGF mouse model showed an increased IOP, decreased a- and b-wave amplitudes via ERG, less RGCs, a macroglia activation, and fewer cone bipolar cells in 15-week-old animals [11].

In the current study, we examined apoptosis and synaptic alterations in these βB1-CTGF mice. With regard to apoptotic mechanisms, we noted more cleaved caspase 3^+^ apoptotic RGCs in βB1-CTGF eyes as well as an upregulation of *Casp3* mRNA levels. Moreover, TUNEL staining revealed more apoptotic cells in the ganglion cell layer of transgenic mice at 5 and 10 weeks of age. In an experimental autoimmune glaucoma (EAG) model, a higher number of caspase 3^+^ cells was observed 14 days after induction, while a significant loss of RGCs occurred after 22 days IOP-independently [21]. Furthermore, in other ocular hypertension (OHT) studies, apoptosis was observed via activation of the caspase pathway in the retina. For example, more cleaved caspase 3^+^ cells could be detected in 5-month-old DBA/2J mice and a RGC loss was detected at 9 months [22]. This timeline, apoptosis followed by RGC loss, is in accordance with our results.

Furthermore, the mRNA levels of *Bax/Bcl2* were significantly upregulated in 10-week-old βB1-CTGF mice. BAX is a proapoptotic member of the BCL2 family and plays a major role in mitochondrial-mediated apoptosis in different neuronal cells [23,24]. It was shown that deficiency of BAX prevented RGC degeneration in DBA/2J mice [25]. These results lead to the assumption that retinal degeneration occurred through apoptosis in these models. Additionally, in glaucoma patients, studies indicate that RGCs die via apoptotic pathways [26].

We further investigated RGCs and neurofilament H via immunohistology and RT-qPCR analyses. We could see similar RGC numbers and *Pou4f1* mRNA expression levels in βB1-CTGF animals compared to in WT at 5 and 10 weeks, while a significant downregulation of *Tubb3* mRNA could be revealed in 10-week-old transgenic mice. Furthermore, we observed significantly decreased neurofilament H staining in 10-week-old βB1-CTGF animals. We assume that axonal changes occur before a loss of RGC cell bodies, which is observed in 15-week-old transgenic mice [11]. In a study using focal ischemia in pig eyes, the authors observed that neurofilament H was one of the earliest cytoskeleton subunits that underwent change [27].

Hence, in βB1-CTGF mice, early apoptosis triggers subsequent cell loss, which not only is similar to the progression in DBA/2J mice but has also some similarities to progressive damage in patients. In glaucoma patients, RGC death and visual field loss are known to occur slowly over time and apoptosis is revealed as an early marker of this disease [3,4,6,28].

The response of RGCs to visual stimuli is a result of signal integration from all cell compartments, namely cell body, axon, dendrites, and synaptic terminals [29]. However, only a few studies analyzed the role of synapses in glaucoma. In the EAG model, a loss of RGCs was observed, which was accompanied by degeneration of the presynaptic active zone protein bassoon and the postsynaptic protein PSD-95 28 days after immunization [30]. Furthermore, mice immunized with an optic nerve homogenate displayed fewer glutamatergic and GABAergic synapses after 6 weeks [31]. In DBA/2J mice, a loss of synaptic contacts was found in an aged-related manner. Fernandez-Sanchez et al. observed an absence of Vglut1 immunoreactivity in photoreceptor ribbons in 16-month-old mice [32]. In our study presented here, we noted a decrease in the gephyrin^+^ signal intensity in transgenic animals at 5 weeks. Interestingly, at 10 weeks, the intensity of gephyrin reverted to the WT level although a downregulation of the corresponding gene was observed via RT-qPCR.

In Alzheimer’s disease, not only does neuronal degeneration occur but also another hallmark is a loss of synapses [33,34]. It is known that, at first, neuronal and synaptic dysfunction is detectable, which triggers a compensatory response of synapses to maintain synaptic connectivity. New synapses are formed, and the remaining synapses gain size [35,36,37]. Secondly, cycles of aberrant sprouting and neuritic disorganization lead to synapse loss and neurodegeneration [35,38,39]. Additionally, in an OHT glaucoma model, synaptic vesicle proteins first increased one week after IOP elevation and, then, the total number of ribbon synapses decreased after 8 weeks. However, the authors observed immature and newly formed ribbon synapses via electron microscopy, indicating compensatory mechanisms to restore synaptic connections [40]. We therefore assume that similar mechanisms could also occur in regard to GABAergic synapses in our model. The increase in synaptic proteins could be used as compensatory factors to maintain visual integrity. In contrast to gephyrin, Vglut1 intensity was decreased at both investigated ages in βB1-CTGF mice. Vglut1 in the retina is necessary for synaptic signaling of visual-evoked responses from photoreceptors cells [41]. In diabetic rat retinae, a downregulation of Vglut1 was detected on protein as well as gene levels [42]. In the EAG model, a downregulation of *Slc1717* mRNA levels could be revealed 6 weeks after immunization [31]. The loss of Vglut1 intensity might indicate changes in the transmission of signals. However, more investigations are needed to gain additional information about synaptic dysfunction in this model.

We also investigated other cell types in the retina, bipolar cells, and photoreceptors to examine whether they are affected in this model. To detect the number of bipolar cells, we used PKCα, which plays an important role in the modulation of aqueous outflow facility [43], as a rod bipolar cell marker and recoverin as a cone bipolar cell marker [44]. The numbers of rod and cone bipolar cells as well as the corresponding mRNA levels remained unchanged in 5- and 10-week-old βB1-CTGF mice. At 15 weeks of age, a diminished amount of cone bipolar cells could be noted in this model [11]. Studies using the DBA/2J mice demonstrated similar results regarding the loss of bipolar cells at young ages. Here, the first decline in bipolar cells occurred after 3 months [32,45].

Regarding investigations of photoreceptors, we could not note any difference in rods and L-cones between βB1-CTGF and wildtype mice at the age of 5 and 10 weeks. It is known that photoreceptors are eventually affected by chronically elevated IOP [46]. While some studies show that the cone density of glaucoma patients does not differ significantly from healthy eyes [47,48], cone photoreceptors seem to be affected in advanced cases of glaucoma [49]. In DBA/2J mice, degeneration of photoreceptors at 3 months was noted [32], while in an OHT rat model, animals developed a loss of cones after only 4 weeks [50]. This could be explained by the different methods of inducing ocular hypertension. In this case, they used laser coagulation of the trabecular meshwork, the perilimbal, and episcleral vessels, which induces more damage compared to a nonsurgical method. In the βB1-CTGF mouse model, where IOP increases gradually over time, the photoreceptors seem to not be affected at young ages, making it a useful model to study the slowly progressing glaucoma damage.

In glaucoma, macroglia show a reactive and not proliferative response causing remodeling processes [51,52,53]. Hence, we used anti-GFAP and anti-glutamine synthetase antibodies as astrocyte and Müller glia markers to detect possible gliosis. It is known that glutamine synthetase is overexpressed in aqueous humor of glaucoma patients [54]. In our study, we did not see gliosis after 5 and 10 weeks. In a previous study, activation of macroglia was seen in older βB1-CTGF mice (15 weeks old) [11]. In addition, in DBA/2J mice, a gliosis could not be detected until 3 months of age [51,55]. In a rat OHT study, Müller cell gliosis was observable after only one week [56]. Nonetheless, it is difficult to compare these results with ours, since early macrogliosis could be caused by the surgical intervention in these rats. In contrast, damage in the DBA/2J as well as the βB1-CTGF model occurs without surgical procedures. However, not only in animal studies but also in glaucoma patients, macrogliosis is detectable. Ashimatey et al. noted hyperreflective structures in the OCT of glaucoma patients, which were presumed to be activated astrocytes and Müller cells [57]. Furthermore, patients with a high number of retinal astrocytes and Müller cells revealed lower retinal sensitivity and reduced nerve fiber thickness in an advanced POAG disease stage [58]. In contrast, healthy persons did not show any activation of macroglia [59]. Therefore, these studies presume that activated astrocytes and Müller cells could be a clinical sign for advanced stages of POAG, but not for early ones. In addition, in βB1-CTG mice, astrogliosis seems to occur only in advanced stages, when RGC loss is noted [11].

## 4. Materials and Methods

### 4.1. Animals

All procedures concerning animals adhered to the ARVO statement for the use of animals in ophthalmic and vision research. All experiments involving animals were approved by the animal care committee of North Rhine-Westphalia, Germany (approval codes: 84-02.04.2013.A291 and 81-02.04.218.A071). Mice were kept under environmentally controlled conditions with free access to chow and water.

The used transgenic βB1-CTGF and WT mice in this study had a CD1 background [11]. The WT CD1 mice for breeding were obtained from Charles River (Sulzfeld, Germany), while the βB1-CTGF mice for breeding were kindly provided by Prof. Dr. Fuchshofer (University Regensburg, Germany). Then, all animals for this study were bred and housed at the animal facility at the Ruhr-University Bochum (Bochum, Germany). Potential βB1-CTGF mice were screened by isolating genomic DNA from tail biopsies and testing for transgenic sequenced by PCR using the following primer sequences: 5′-GGAAGTGCCAGCTCATCAGT-3’ and 5´- GTGCGGGACAGAAACCTG-3´. 5- and 10-week-old female and male mice were included in the study.

### 4.2. Optical Coherence Tomography

Mice were anesthetized with ketamine (120 mg/kg) and xylazine (16 mg/kg). SD-OCT images were captured using Spectralis HRA + OCT (Heidelberg Engineering GmbH, Heidelberg, Germany) in 5- and 10-week-old βB1-CTGF and WT mice (n = 6–8 animals/group) [60,61]. To measure the retinal thickness, central and peripheral images were taken with a 30-degree OCT field. The total retinal thickness as well as the thickness of the ganglion cell complex (nerve fiber layer, ganglion cell layer, and inner plexiform layer) was measured in an axis perpendicular to the individual layers using ImageJ software (NIH; Bethesda, MD, USA). Seven measurements were used to calculate the mean value for each eye [62]. At the end, the means of both groups were compared for the central and peripheral parts of the retina as well as for the total retina thickness.

### 4.3. Electroretinogram Analyses

For ERG measurements, mice were dark adapted overnight. The retinal function was monitored using full-field-flash electroretinography (HMsERG system, OcuScience, Henderson, NV, USA) in 5- and 10-week-old mice of both groups (n = 6–8 animals/group) [11]. The mice were anesthetized with a ketamine/xylazine cocktail (120/16 mg/kg), and their eyes were dilated with tropicamide (5%) and topically anesthetized using conjuncain (Bausch&Lomb, Berlin, Germany). Body temperature was maintained at 37 °C with a feedback temperature controller (TC-1000; CWE Inc., Ardmore, PA, USA). Reference electrodes were placed subcutaneously below the right and left ears, and a ground electrode was placed at the base of the tail. Contact lenses with silver thread recording electrodes were placed in the center of the cornea after the application of methocel (Omni Vision, Puchheim, Germany). Before measurement, the electroretinography equipment was covered with a Faraday cage. Scotopic flash ERGs were recorded at 0.1, 0.3, 1, 3, 10, and 25 cd/m^2^. The signals obtained from the corneal surface were amplified, digitized, averaged, and stored using commercial software (ERGView 4.380R; OcuScience). A 50 Hz filtering of the data was applied before evaluating the amplitude of the a- and b-waves.

### 4.4. Tissue Preparation for Immunohistology

After 5 and 10 weeks, mice eyes were enucleated and fixed in 4% paraformaldehyde for 1 h. Thereafter, the eyes underwent a 10% sucrose treatment and were embedded in a Neg-50 compound (Tissue-Tek; Fisher Scientific, Schwerte, Germany). Ten micrometer thick cross sections were cut with a cryostat (Fisher Scientific) for further staining [63].

### 4.5. Immunohistology

In order to identify different retinal cell types, specific immunofluorescence antibodies were applied (*n* = 5–9 eyes/group; 6 sections/animal, Table 1) [64]. Briefly, retinal cross sections were blocked with a solution containing 10–20% donkey, 2–3% bovine serum albumin, and/or goat serum and 0.1% Triton-X in PBS. The sections were incubated with primary antibodies at room temperature overnight. Incubation using corresponding secondary antibodies was performed for 1 h on the next day. For the detection of apoptotic cells, a commercially available TUNEL Kit (In Situ Cell Death Detection Kit; Roche; Sigma-Aldrich, St. Louis, MO, USA) was used according to the manufacturer’s instructions. Nuclear staining with 4′,6 diamidino-2-phenylindole (DAPI, Serva Electrophoresis, Heidelberg, Germany) was included to facilitate orientation on the slides. Negative controls were performed for each stain by using secondary antibodies only. 

### 4.6. Histological Examination

The photographs were taken using a fluorescence microscope (Axio Imager M1 or M2, Zeiss, Jena, Germany). Two photos of the peripheral and two of the central part of each section were captured at distances 300 and 3100 µm dorsal and ventral to the optic nerve for each retinal cross section (6 sections/eye, n = 5–9 eyes/group) [30,65]. The images were transferred to Corel Paint Shop Pro (V13, Corel Corporation, Ottawa, ONT, Canada) and equal excerpts of 800 × 600 pixel (respectively, 700 × 300 pixel for neurofilament H) were cut out. Afterwards, Brn-3a^+^, PKCα^+^, opsin^+^, and TUNEL^+^ cells were counted in a masked fashion using ImageJ software [11]. Regarding GFAP, glutamine synthetase, rhodopsin, recoverin, and neurofilament H, the percentage of stained area was analyzed using ImageJ software. With regard to gephyrin and Vglut1 staining, the staining intensity was evaluated. For both ImageJ analyses, the images were transformed into grayscale. To minimize interference with background labeling, a defined rolling ball radius was subtracted (Table 2). Then, for each picture, suitable lower and upper thresholds were defined. The ideal values were obtained when the grayscale picture corresponded to the original one. Once the thresholds from all pictures were set, the mean value for the lower threshold was calculated, and this number was used for final analyses. The percentage (respectively, the intensity) of the stained area was then measured between these defined threshold (Table 2) [63,66].

### 4.7. Quantitative Real-Time PCR

Both retinae of each animal (n = 5–7 animals/group) were pooled for RNA preparation and cDNA synthesis as previously described [11]. The designed oligonucleotides for RT-qPCR are shown in Table 3. Βeta-actin (*Actb*) and cyclophilin (*Ppid*) served as reference genes. The RT-qPCR was performed using DyNAmo Flash SYBR Green (Fisher Scientific) on the PikoReal RT-qPCR Cycler (Fisher Scientific) [67,68]. The values were transferred to REST© software (Qiagen, Hilden, Germany) for further analysis.

### 4.8. Statistics

SD-OCT, ERG, and immunohistological data are presented as mean ± SEM. The βB1-CTGF animals were compared to the WT group via two-tailed Student’s t-test using Statistica Software (Version 13, Dell, Tulsa, OK, USA). For SD-OCT and immunohistology, the WT values were set to 100%. Cleaved caspase 3^+^ and TUNEL^+^ cells are displayed as cells/mm. Regarding RT-qPCR, the relative expression values are presented as median ± quartile + minimum/maximum and were assessed via Pair Wise Fixed Reallocation Randomization Test© using REST© software (Qiagen) [69]. *p*-values below 0.05 were considered statistically significant, with * *p* < 0.05, ** *p* < 0.01, and *** *p* < 0.001.

## 5. Conclusions

With this study, we investigated apoptotic mechanisms and synapses in a βB1-CTGF mouse model. We noted more apoptotic RGCs, which was followed by a loss of neurofilament H. This slow progressive degeneration mimics the situation in glaucoma patients. Hence, the results underline that βB1-CTGF mice serve as a suitable model to study neurodegeneration occurring in POAG in more detail.

## Figures and Tables

**Table 1:** Primary and secondary antibodies used for immunohistology.

Primary Antibodies			Secondary Antibodies		
Antibody	Company	Dilution	Antibody	Company	Dilution
Anti-Brn3a	Santa Cruz	1:100	Donkey anti-goat Alexa Fluor 488	Dianova	1:500
Anti-cleaved caspase 3	Sigma-Aldrich	1:300	Donkey anti-rabbit Alexa Fluor 555	Invitrogen	1:500
Anti-gephyrin	Synaptic Systems	1:500	Donkey anti-rabbit Alexa Fluor 488	Thermo Fisher	1:500
Anti-GFAP	Millipore	1:500	Donkey anti-chicken Cy3	Millipore	1:500
Anti-glutamine synthetase	Abcam	1:500	Goat anti-mouse Alexa Fluor 488	Invitrogen	1:500
Anti-neurofilament H	Synaptic Systems	1:500	Donkey anti-chicken Cy3	Millipore	1:500
Anti-opsin	Millipore	1:1200	Donkey anti-rabbit Alexa Fluor 555	Invitrogen	1:500
Anti-PKCα	Santa Cruz	1:300	Goat anti-mouse Alexa Fluor 488	Invitrogen	1:500
Anti-rhodopsin	Abcam	1:400	Goat anti-mouse Alexa Fluor 488	Eugene	1:500
Anti-ribeye	Synaptic Systems	1:500	Donkey anti-guinea pig Alexa Fluor 488	Jackson Immuno Research	1:500
Anti-recoverin	Millipore	1:1000	Donkey anti-rabbit A555	Invitrogen	1:400
Anti-Vglut1	Synaptic Systems	1:500	Donkey anti-chicken Cy3	Millipore	1:500
Anti-rhodopsin	Abcam	1:400	Goat anti-mouse Alexa Fluor 488	Eugene	1:500

**Table 2:** Adjustments in ImageJ macro for area analysis. The background subtraction (pixel) as well as the lower and the upper thresholds are listed.

Protein	Background	Lower Threshold	Upper Threshold
Gephyrin	50	6.01	252.22
GFAP	50	8.97	246.72
Glutamine synthetase	50	10.47	262.82
Neurofilament H	50	10.22	84.63
Recoverin	50	9.95	264.02
Rhodopsin	50	9.02	265.00
Vglut1	50	6.62	250.29

**Table 3:** Sequences of oligonucleotides. The listed oligonucleotide pairs were used in quantitative real-time PCR experiments, while Βeta-actin (Actb) and Cyclophilin (Ppid) served as housekeeping genes. The predicted amplicon sizes are given. Abbreviations: F = forward, R = reverse, acc. no. = accession number, bp = base pair.

Gene	Forward (F) and Reverse (R) Oligonucleotides	GenBank Accession Number	Amplicon Size
*Actb*-F *Actb*-R	ctaaggccaaccgtgaaag accagaggcatacagggaca	NM_007393.5	104 bp
*Bax*-F *Bax-R*	gtgagcggctgcttgtct gtgggggtcccgaagtag	NM_007527.3	73 bp
*Bcl2*-F *Bcl2*-R	agtacctgaaccggcatctg ggggccatatagttccacaaa	NM_009741.5	77 bp
*Casp3*-F *Casp3*-R	gaggctgacttcctgtatgctt aaccacgacccgtccttt	NM_001284409.1	77 bp
*Gphn*-F *Gphn*-R	tgatcttcatgctcagatcca ttgcaaatgttgttggcaag	NM_145965.2	68 bp
*Nefh*-F *Nefh*-R	cattgagattgccgcttaca actcggaccaaagccaatc	NM_010904.3	67 bp
*Pou4f1*-F *Pou4f1*-R	ctccctgagcacaagtaccc ctggcgaagaggttgctc	AY706205.1	98 bp
*Ppid*-F *Ppid*-R	ttcttcataaccacaagtcaagacc tccacctccgtaccacatc	M60456.1	95 bp
*Prkca*-F *Prkca*-R	caagggatgaaatgtgacacc cctcttctctgtgtgatccattc	NM_011101.3	96 bp
*Rcvrn*-F *Rcvrn*-R	caatgggaccatcagcaaa cctaggcttgatcattttga	NM_009038.2	71 bp
*Rho*-F *Rho*-R	tgtggtcttcacctggatcat gaacattgcatgccctcag	NM_145383.1	90 bp
*Slc17a7*-F *Slc17a7*-R	gtgcaatgaccaaggacaag agatgacaccgccgtagtg	NM_182993.2	103 bp
*Slc18a3*-F *Slc18a3*-R	agagccctaccctgatctctg caagtaggcgctggcattag	NM_021712.3	77 bp

## Data Availability

Data sharing not applicable.

## Supplementary Materials

The following are available online at https://www.mdpi.com/1422-0067/22/4/1997/s1, Figure S1: No functional or morphological alterations.
