# Supplementary material for: Activation of Apoptosis in a βB1-CTGF Transgenic Mouse Model
Source: Int J Mol Sci. 2021 Feb 17;22(4):1997. doi: 10.3390/ijms22041997 (PMC7922353; doi:10.3390/ijms22041997)
Supplement: Supplementary file 1 [file ijms-22-01997-s001.pdf]

Activation of apoptosis in a βB1-CTGF glaucoma model

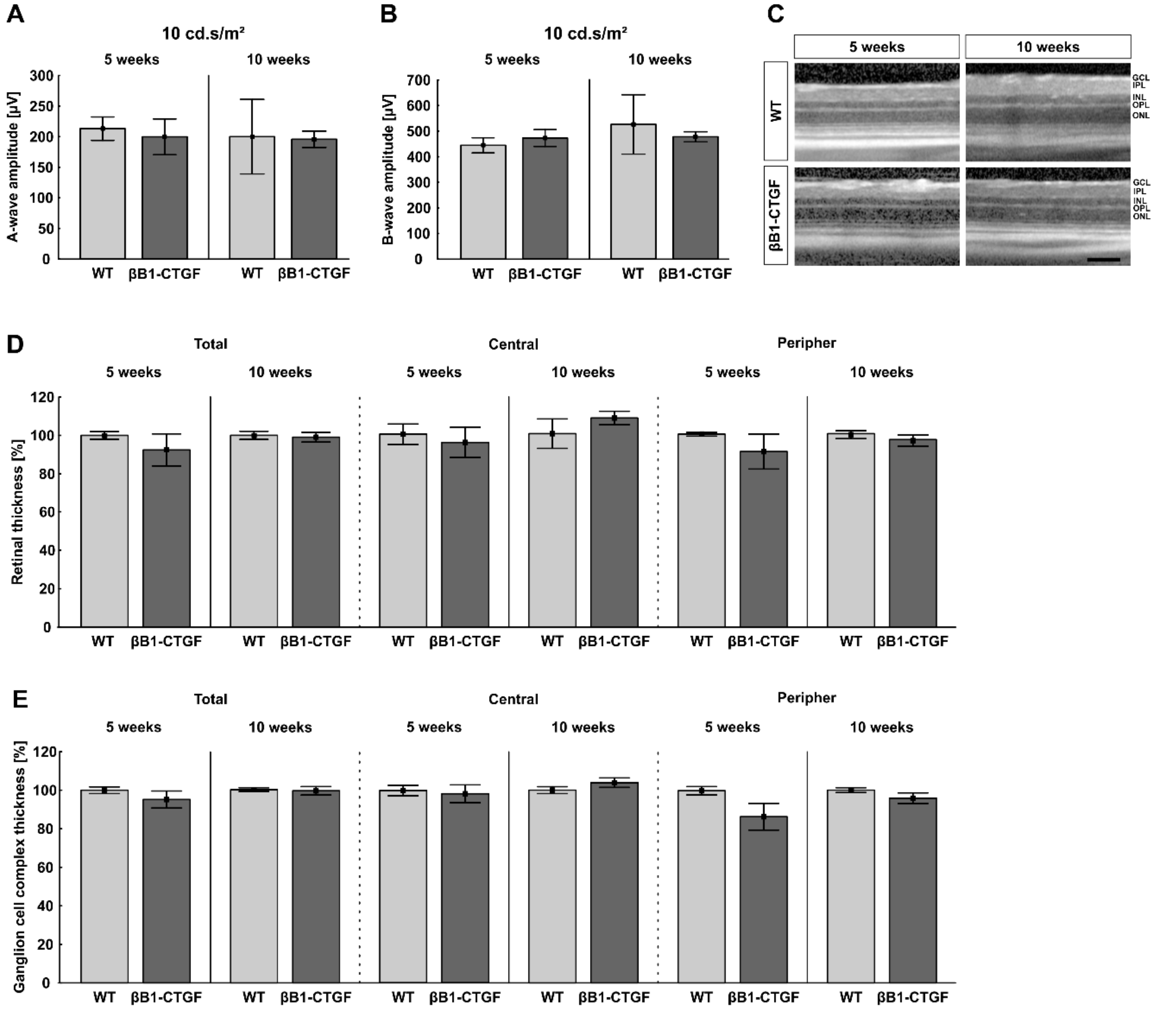

Supplement figure 1: No functional and morphological alterations. **A)** ERG measurements were performed in 5- and 10-week-old mice (n=6-8/group). Exemplarily, the recording at a light intensity of 10 cd.s/m^2 is pictured. The a-wave amplitude was comparable between βB1-CTGF and WT mice at both ages. **B)** No significant change of the b-wave amplitude could be noted in βB1-CTGF mice at 5 and 10 weeks of age. **C)** At 5 and 10 weeks of age, SD-OCT analyses were performed to measure the retinal thickness and the ganglion cell complex thickness in total as well as in central and peripheral parts (n=6-8/group). **D)** The total retinal thickness was comparable between βB1-CTGF and WT mice at 5 and 10 weeks. In addition, at both ages, the central and peripheral retinal thickness was not altered between transgenic and WT animals. **E)** Additionally, the total thickness of the ganglion cell complex did not differ in transgenic mice compared to WT animals at both ages. Moreover, the thickness of the central and peripheral ganglion cell complex remained unaltered in 5- and 10-week-old mice. Abbreviations: GCL=ganglion cell layer, IPL=inner plexiform layer, INL=inner nuclear layer, OPL=outer plexiform layer, ONL=outer nuclear layer. Values are mean±SEM. Scale bar: 200 µm.
